# Supplementary material for: Synthesis of Water Resistance and Moisture-Permeable Nanofiber Using Sodium Alginate–Functionalized Waterborne Polyurethane
Source: Polymers (Basel). 2020 Dec 1;12(12):2882. doi: 10.3390/polym12122882 (PMC7761416; doi:10.3390/polym12122882)
Supplement: Supplementary file 1 [file polymers-12-02882-s001.pdf]

Supporting Information

Synthesis of water resistance and moisture-permeable nanofiber using sodium alginate–functionalized waterborne polyurethane

Wen-Chi Lu,[a,b] Fu-Sheng Chuang,[c] Manikandan Venkatesan,[a] Chia-Jung Cho,[a,*] Po-Yun Chen,[a] Yung-Ru Tzeng,[a] Yang-Yen Yu,[d] Syang-Peng Rwei,[a] and Chi-Ching Kuo,[a,*]

[a] Institute of Organic and Polymeric Materials, Research and Development Center of Smart Textile Technology, National Taipei University of Technology, Taipei 10608, Taiwan

[b] Department of Applied Cosmetology, Lee-Ming Institute of Technology, New Taipei City 243083, Taiwan

[c] Department of Fashion and Design, Lee-Ming Institute of Technology, New Taipei City 243083, Taiwan

[d] Department of Materials Engineering, Ming Chi University of Technology, New Taipei City, 24301, Taiwan

*Author to whom all correspondence should be addressed

Tel: 886-2-27712171*2407 Fax: 886-2-27317174

Correspondence to: *Prof. C.-C. Kuo: kuocc@mail.ntut.edu.tw

*Dr. C.-J. Cho: ppaul28865@mail.ntut.edu.tw

Table S1. The composition of WPU/SA blends

Code[1]	WPU(wt%)	SA(wt%)
**WPU/SA $_{10}$**	**90**	**10**
**WPU/SA $_{20}$**	**80**	**20**
**WPU/SA $_{30}$**	**70**	**30**
**WPU/SA $_{40}$**	**60**	**40**
**WPU/SA $_{50}$**	**50**	**50**

(1) Sample name ： WPU and SA blended samples were named WPU/SA_{xx}, and the subscripts xx was the weight percentages of SA. Similarly, the samples of WPU/SA_{xx} blends which crosslinking with calcium chloride were named WPU/CA_{yy}.

Table S2. Water absorption of WPU/CA blends

Code	water absorption [1,2](wt%)
**WPU**	**10.2**
**CA**	**139.1**
**WPU/CA_{10}**	**25.7**
**WPU/CA_{20}**	**26.9**
**WPU/CA_{30}**	**37.5**
**WPU/CA_{40}**	**57.3**
**WPU/CA_{50}**	**82.5**

1.Water absorption (wt%) = wet weight – dry weight/dry weight×100

2. All dry films of blends were placed in deionized water of 23 ± 1°C for 2 hours

Table S3. Moisture permeability of WPU/CA

Code	Moisture permeability (g/m^2-24hr)
WPU	6.2
CA	69.1
WPU/CA_{10}	19.0
WPU/CA_{20}	20.0
WPU/CA_{30}	25.1
WPU/CA_{40}	31.4
WPU/CA_{50}	34.7

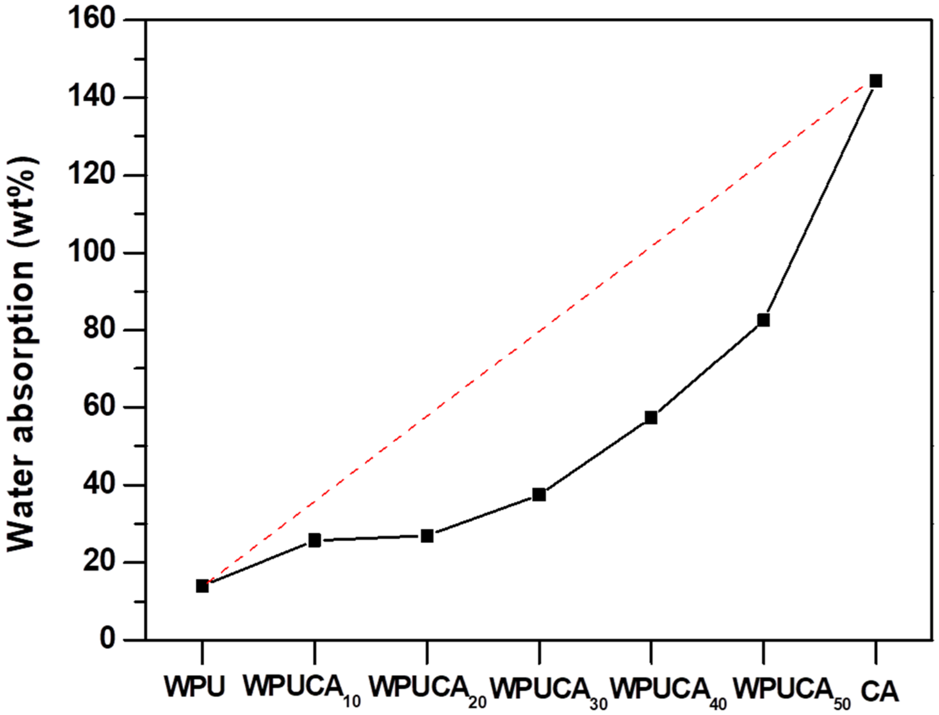

Figure S1. Water absorption during 2hrs of WPU/CA blends

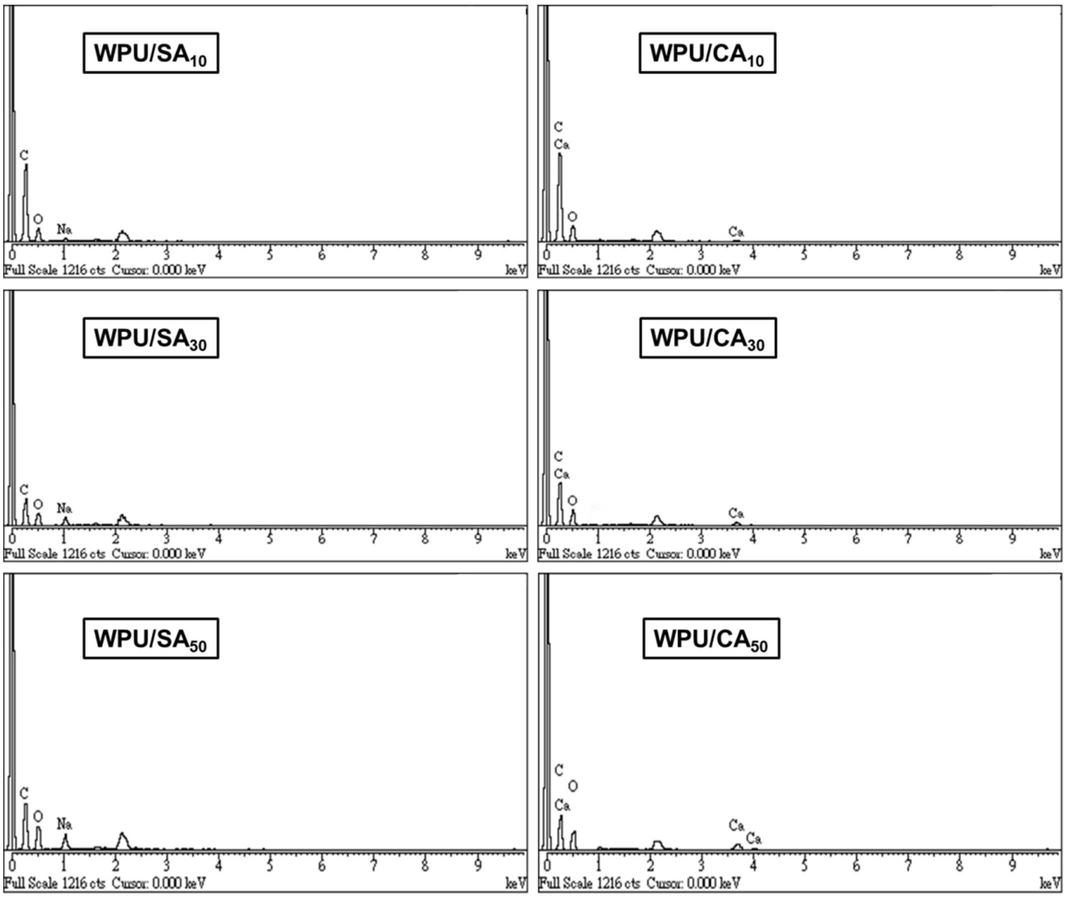

Figure S2. EDS of cross section of WPU/CA blends

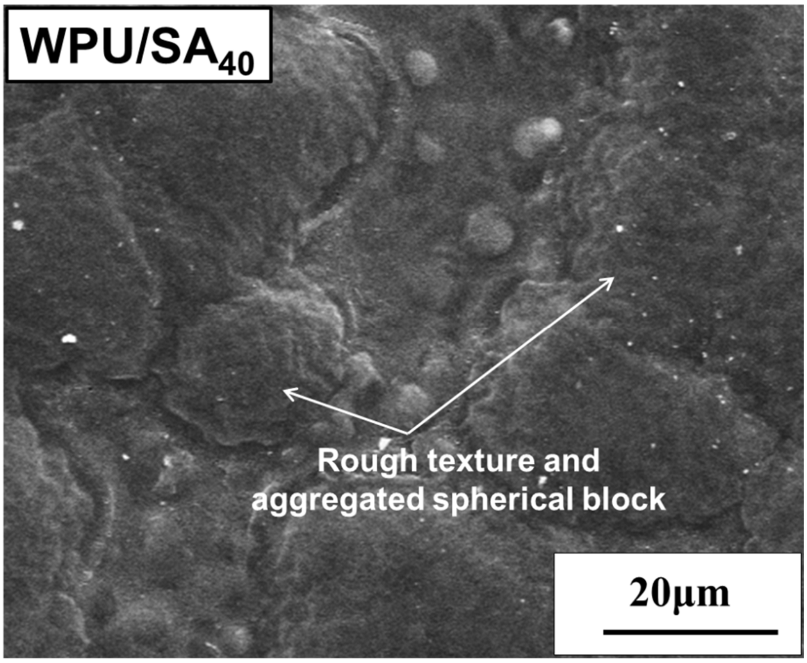

Figure S3. Rough texture and aggregated spherical block of WPU/CA_{40} SEM Surface morphology

Video S1. A lab experiment was carried out to confirm the moisture permeability of

nanofiber web.
